# 1,4-Bis(4-pyridylmeth­oxy)benzene

**DOI:** 10.1107/S1600536809038707

**Published:** 2009-09-30

**Authors:** Ping Zou, Ying Liu, Shuang Zhang, Xue Wang, Jin-Sheng Gao

**Affiliations:** a College of Life Science, Sichuan Agriculture University, Yaan 625014, People’s Republic of China; b College of Chemistry and Materials Science, Heilongjiang University, Harbin 150080, People’s Republic of China

## Abstract

The mol­ecule of the title compound, C_18_H_16_N_2_O_2_, lies about a center of inversion. The central phenyl­ene ring is aligned at 62.7 (1)° with respect to the pyridyl ring. In the crystal, weak inter­molecular C—H⋯N hydrogen bonds link mol­ecules into sheets parallel to (104). C—H⋯O inter­actions are also present.

## Related literature

For general background to metal-organic complexes with flexible pyridyl-based ligands, see: Hou *et al.* (2001 ▶). For details of the synthesis, see Gao *et al.* (2004 ▶). For related structures, see: Gao *et al.* (2006 ▶, 2009*a* ▶,*b* ▶).
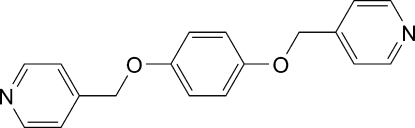


## Experimental

### 

#### Crystal data

C_18_H_16_N_2_O_2_
*M* *_r_* = 292.33
Monoclinic, 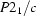

*a* = 6.7825 (14) Å
*b* = 5.8694 (12) Å
*c* = 18.542 (4) Å
β = 90.99 (3)°
*V* = 738.0 (3) Å^3^
*Z* = 2
Mo *K*α radiation
μ = 0.09 mm^−1^
*T* = 291 K
0.22 × 0.17 × 0.15 mm

#### Data collection

Rigaku R-AXIS RAPID diffractometer
Absorption correction: multi-scan (*ABSCOR*; Higashi, 1995 ▶) *T*_min_ = 0.981, *T*_max_ = 0.987
6972 measured reflections
1692 independent reflections
1381 reflections with *I* > 2σ(*I*)
*R*_int_ = 0.025

#### Refinement

*R*[*F* ^2^ > 2σ(*F* ^2^)] = 0.043
*wR*(*F* ^2^) = 0.123
*S* = 1.09
1692 reflections
100 parameters
H-atom parameters constrained
Δρ_max_ = 0.25 e Å^−3^
Δρ_min_ = −0.22 e Å^−3^

### 

Data collection: *RAPID-AUTO* (Rigaku, 1998 ▶); cell refinement: *RAPID-AUTO*; data reduction: *CrystalClear* (Rigaku/MSC, 2002 ▶); program(s) used to solve structure: *SHELXS97* (Sheldrick, 2008 ▶); program(s) used to refine structure: *SHELXL97* (Sheldrick, 2008 ▶); molecular graphics: *SHELXTL* (Sheldrick, 2008 ▶); software used to prepare material for publication: *SHELXL97*.

## Supplementary Material

Crystal structure: contains datablocks I, global. DOI: 10.1107/S1600536809038707/ng2651sup1.cif

Structure factors: contains datablocks I. DOI: 10.1107/S1600536809038707/ng2651Isup2.hkl

Additional supplementary materials:  crystallographic information; 3D view; checkCIF report

## Figures and Tables

**Table 1:** Hydrogen-bond geometry (Å, °)

*D*—H⋯*A*	*D*—H	H⋯*A*	*D*⋯*A*	*D*—H⋯*A*
C5—H5⋯N1^i^	0.93	2.62	3.4499 (18)	149
C9—H9⋯O1^ii^	0.93	2.63	3.5156 (17)	160

Symmetry codes: (i) 
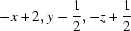
; (ii) 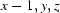.

**Fig. 1.**
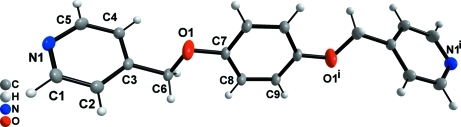
The molecular structure of the title compound, showing displacement ellipsoids at the 50% probability level for non-H atoms. [Symmetry codes: (i) -x, -y, 1 - z]

**Fig. 2.**
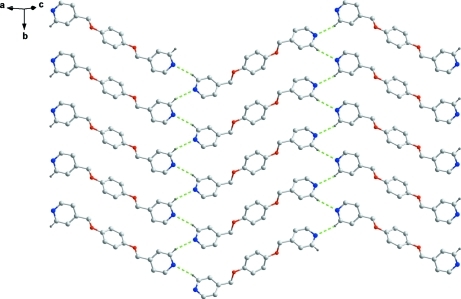
A partial packing view, showing the two-dimensional hydrogen bonding sheet. Dashed lines indicate the hydrogen-bonding interactions.

## supplementary crystallographic information

### Comment

The metal-organic complexes with flexible pyridyl-based ligands have been rapidly developed in the past few years owing to their abundant topology structures and potential applications. Hou's group (2001) has reported the synthesis of 1,4-bis(4-pyridylmethoxy)benzene ligand, which reacted with Cd(NO_3_)_2_.6H_2_O and Co(NCS)_2_ to assemble into one-dimensional chain and two-dimensional plane network structures, respectively. It is worthy to note that the former cadmium complex consists of two kinds of chains: double zigzag linear chain and ladder-like one-dimensional chain. Our group has report three kinds of flexible pyridyl-based ligands in the previous report (Gao *et al.* 2006; Gao *et al.* 2009*a*; Gao *et al.* 2009*b*). As an extension of our work about bipyridyl aromatic ligands, we have synthesized and report the crystal structure of the title compound here.

In the title compound, the 1,4-bis(4-pyridylmethoxy)benzene ligand is centrosymmetric. The planes of two terminal pyridyl groups distort drastically and have dihedral angles of 62.7 (1)62.7 (1)° with the plane of the central benzene ring (Figure 1).

Within the packing structure,the adjacent 1,4-bis(4-pyridylmethoxy)benzene molecules are linked into two-dimensional wavy structure in (104) direction by intermolecular C—H···N hydrogen bonds interactions existing in the terminal pyridine rings (Figure 2, Table 1).

### Experimental

The 1,4-bis(4-pyridylmethoxy)benzene was synthesized by the reaction of *p*-benzenediol and 4-chloromethylpyridine hydrochloride under nitrogen atmosphere and alkaline condition (Gao *et al.*, 2004; Gao *et al.*, 2006). Colourless block-shaped crystals of title compound were obtained by slow evaporation of an methanol solution after several days.

### Refinement

H atoms bound to C atoms were placed in calculated positions and treated as riding on their parent atoms, with C—H = 0.93 Å (aromatic), C—H = 0.97 Å (methylene), and with *U*_iso_(H) = 1.2*U*_eq_(C).

### Crystal data

**Table:**

C_18_H_16_N_2_O_2_	*F*(000) = 308
*M**_r_* = 292.33	*D*_x_ = 1.315 Mg m^−^^3^
Monoclinic, *P*2_1_/*c*	Mo *K*α radiation, λ = 0.71073 Å
Hall symbol: -P 2ybc	Cell parameters from 5914 reflections
*a* = 6.7825 (14) Å	θ = 3.0–27.5°
*b* = 5.8694 (12) Å	µ = 0.09 mm^−^^1^
*c* = 18.542 (4) Å	*T* = 291 K
β = 90.99 (3)°	Block, colorless
*V* = 738.0 (3) Å^3^	0.22 × 0.17 × 0.15 mm
*Z* = 2	

### Data collection

**Table:**

Rigaku R-AXIS RAPID diffractometer	1692 independent reflections
Radiation source: fine-focus sealed tube	1381 reflections with *I* > 2σ(*I*)
graphite	*R*_int_ = 0.025
ω scans	θ_max_ = 27.5°, θ_min_ = 3.0°
Absorption correction: multi-scan (*ABSCOR*; Higashi, 1995)	*h* = −8→8
*T*_min_ = 0.981, *T*_max_ = 0.987	*k* = −7→7
6972 measured reflections	*l* = −24→23

### Refinement

**Table:**

Refinement on *F*^2^	Primary atom site location: structure-invariant direct methods
Least-squares matrix: full	Secondary atom site location: difference Fourier map
*R*[*F*^2^ > 2σ(*F*^2^)] = 0.043	Hydrogen site location: inferred from neighbouring sites
*wR*(*F*^2^) = 0.123	H-atom parameters constrained
*S* = 1.09	*w* = 1/[σ^2^(*F*_o_^2^) + (0.0725*P*)^2^ + 0.0696*P*] where *P* = (*F*_o_^2^ + 2*F*_c_^2^)/3
1692 reflections	(Δ/σ)_max_ < 0.001
100 parameters	Δρ_max_ = 0.25 e Å^−^^3^
0 restraints	Δρ_min_ = −0.22 e Å^−^^3^

### Special details

**Table:**

Geometry. All e.s.d.'s (except the e.s.d. in the dihedral angle between two l.s. planes) are estimated using the full covariance matrix. The cell e.s.d.'s are taken into account individually in the estimation of e.s.d.'s in distances, angles and torsion angles; correlations between e.s.d.'s in cell parameters are only used when they are defined by crystal symmetry. An approximate (isotropic) treatment of cell e.s.d.'s is used for estimating e.s.d.'s involving l.s. planes.
Refinement. Refinement of *F*^2^ against ALL reflections. The weighted *R*-factor *wR* and goodness of fit *S* are based on *F*^2^, conventional *R*-factors *R* are based on *F*, with *F* set to zero for negative *F*^2^. The threshold expression of *F*^2^ > σ(*F*^2^) is used only for calculating *R*-factors(gt) *etc*. and is not relevant to the choice of reflections for refinement. *R*-factors based on *F*^2^ are statistically about twice as large as those based on *F*, and *R*- factors based on ALL data will be even larger.

### Fractional atomic coordinates and isotropic or equivalent isotropic displacement parameters (Å^2^)

**Table:**

	*x*	*y*	*z*	*U*_iso_*/*U*_eq_	
C1	0.7686 (2)	0.6857 (2)	0.33856 (8)	0.0480 (4)	
H1	0.8310	0.8236	0.3485	0.058*	
C2	0.5902 (2)	0.6432 (2)	0.37093 (7)	0.0432 (3)	
H2	0.5355	0.7500	0.4018	0.052*	
C3	0.49406 (16)	0.43997 (19)	0.35690 (6)	0.0328 (3)	
C4	0.58453 (18)	0.2877 (2)	0.31094 (6)	0.0382 (3)	
H4	0.5253	0.1487	0.3002	0.046*	
C5	0.76321 (19)	0.3441 (2)	0.28130 (7)	0.0438 (3)	
H5	0.8221	0.2394	0.2508	0.053*	
C6	0.29658 (18)	0.3853 (2)	0.38841 (7)	0.0416 (3)	
H6A	0.2471	0.5155	0.4148	0.050*	
H6B	0.2023	0.3475	0.3503	0.050*	
C7	0.15772 (16)	0.1050 (2)	0.46653 (6)	0.0336 (3)	
C8	−0.03138 (17)	0.1943 (2)	0.45833 (6)	0.0367 (3)	
H8	−0.0528	0.3245	0.4307	0.044*	
C9	−0.18785 (16)	0.0872 (2)	0.49178 (7)	0.0372 (3)	
H9	−0.3146	0.1457	0.4860	0.045*	
N1	0.85625 (16)	0.5413 (2)	0.29416 (6)	0.0466 (3)	
O1	0.32347 (12)	0.19635 (16)	0.43566 (5)	0.0472 (3)	

### Atomic displacement parameters (Å^2^)

**Table:**

	*U*^11^	*U*^22^	*U*^33^	*U*^12^	*U*^13^	*U*^23^
C1	0.0399 (7)	0.0388 (7)	0.0657 (9)	−0.0104 (5)	0.0077 (6)	0.0038 (6)
C2	0.0412 (7)	0.0387 (6)	0.0501 (7)	−0.0021 (5)	0.0115 (5)	−0.0033 (5)
C3	0.0271 (6)	0.0359 (6)	0.0357 (6)	−0.0002 (4)	0.0062 (4)	0.0068 (5)
C4	0.0342 (6)	0.0350 (6)	0.0455 (7)	−0.0027 (5)	0.0060 (5)	0.0000 (5)
C5	0.0362 (7)	0.0479 (7)	0.0476 (7)	0.0044 (5)	0.0127 (5)	−0.0008 (6)
C6	0.0304 (6)	0.0433 (7)	0.0515 (7)	0.0007 (5)	0.0128 (5)	0.0121 (6)
C7	0.0233 (5)	0.0431 (6)	0.0344 (6)	−0.0040 (4)	0.0052 (4)	0.0048 (5)
C8	0.0282 (6)	0.0422 (6)	0.0400 (6)	0.0007 (5)	0.0048 (5)	0.0111 (5)
C9	0.0214 (5)	0.0483 (7)	0.0421 (6)	0.0020 (4)	0.0045 (4)	0.0091 (5)
N1	0.0320 (6)	0.0504 (7)	0.0578 (7)	−0.0028 (4)	0.0127 (5)	0.0106 (5)
O1	0.0229 (4)	0.0619 (6)	0.0572 (6)	−0.0021 (4)	0.0080 (4)	0.0273 (5)

### Geometric parameters (Å, °)

**Table:**

C1—N1	1.3293 (18)	C6—O1	1.4231 (15)
C1—C2	1.3829 (19)	C6—H6A	0.9700
C1—H1	0.9300	C6—H6B	0.9700
C2—C3	1.3818 (17)	C7—O1	1.3789 (13)
C2—H2	0.9300	C7—C9^i^	1.3806 (17)
C3—C4	1.3854 (16)	C7—C8	1.3914 (16)
C3—C6	1.5051 (15)	C8—C9	1.3888 (16)
C4—C5	1.3795 (17)	C8—H8	0.9300
C4—H4	0.9300	C9—C7^i^	1.3806 (17)
C5—N1	1.3379 (18)	C9—H9	0.9300
C5—H5	0.9300		
			
N1—C1—C2	123.89 (12)	O1—C6—H6A	110.2
N1—C1—H1	118.1	C3—C6—H6A	110.2
C2—C1—H1	118.1	O1—C6—H6B	110.2
C3—C2—C1	119.20 (12)	C3—C6—H6B	110.2
C3—C2—H2	120.4	H6A—C6—H6B	108.5
C1—C2—H2	120.4	O1—C7—C9^i^	115.87 (10)
C2—C3—C4	117.39 (11)	O1—C7—C8	124.38 (11)
C2—C3—C6	122.08 (11)	C9^i^—C7—C8	119.75 (10)
C4—C3—C6	120.52 (11)	C9—C8—C7	119.39 (11)
C5—C4—C3	119.47 (11)	C9—C8—H8	120.3
C5—C4—H4	120.3	C7—C8—H8	120.3
C3—C4—H4	120.3	C7^i^—C9—C8	120.86 (11)
N1—C5—C4	123.45 (12)	C7^i^—C9—H9	119.6
N1—C5—H5	118.3	C8—C9—H9	119.6
C4—C5—H5	118.3	C1—N1—C5	116.60 (11)
O1—C6—C3	107.44 (10)	C7—O1—C6	117.51 (9)

Symmetry codes: (i) −*x*, −*y*, −*z*+1.

### Hydrogen-bond geometry (Å, °)

**Table:**

*D*—H···*A*	*D*—H	H···*A*	*D*···*A*	*D*—H···*A*
C5—H5···N1^ii^	0.93	2.62	3.4499 (18)	149
C9—H9···O1^iii^	0.93	2.63	3.5156 (17)	160

Symmetry codes: (ii) −*x*+2, *y*−1/2, −*z*+1/2; (iii) *x*−1, *y*, *z*.
